# Quantifying the biological impacts of nightlights: implications for sleep and circadian health in children

**DOI:** 10.1038/s44323-026-00072-6

**Published:** 2026-04-02

**Authors:** Gena L. Glickman, Rebecca Rothstein-Epp, Kyle Binkowski, Sebastian M. M. Preilipper, Ava Santiago, Sara C. Bessman, Alexandra Easterling, Elizabeth M. Harrison

**Affiliations:** 1 Uniformed Services University of the Health Sciences, Bethesda, MD USA; 2 Bryn Mawr College, Bryn Mawr, PA USA; 3 Henry Jackson Foundation for the Advancement of Military Medicine Inc., Bethesda, MD USA; 4 Arizona State University, Tempe, AZ USA

**Keywords:** Health care, Neuroscience, Physiology

## Abstract

Given the widespread use of nightlights, we characterized an assortment of popular products to evaluate their potential to disrupt sleep and circadian rhythms in children. Spectral irradiance was measured for 25 top-rated nightlights (79 settings) under laboratory (source-level) and simulated bedroom (typical use) conditions. Nightlights varied in form (handheld, projector, tabletop, wall plug-in) as well as intensity and color settings. Melanopic equivalent daylight illuminance (EDI) estimated circadian input and was compared against biologically relevant thresholds for pediatric sleep. Across conditions, melanopic EDI values spanned several orders of magnitude, from 0 lx to >100,000 lx, with some devices varying substantially by setting. With source-level characterizations, only 3/25 products consistently remained below 5 lx melanopic EDI (levels known to phase shift circadian rhythms and suppress melatonin in children), and 2 also met the <1 lx consensus nighttime recommendation for adults. Under typical-use conditions, 14/25 products remained below 5 lx melanopic EDI (11 were <1 lx) on at least one setting, with most being red-toned, low-intensity, and positioned farther from the bed, though many of those would not support vision. Clear clinical guidance, public health recommendations, and improved product labeling standards are needed to reduce circadian disruption and promote healthier sleep in children.

## Introduction

Children are routinely exposed to nighttime light levels far exceeding those found in nature, with significant implications for sleep and circadian health. Nighttime illumination from natural sources, such as moonlight and stars, typically measures below 1 lux (lx), several orders of magnitude dimmer than direct sunlight. The human circadian system evolved under these stark day-night contrasts, yet modern electric lighting disrupts natural cues, contributing to adverse health outcomes, including sleep disturbance, mood disorders, and metabolic dysfunction^1^.

Many children spend daytime hours indoors under electric lighting and remain exposed to ambient light well into the evening. Evening light exposure, particularly from electronic screens (typically 10–100 lx), has been linked to delayed sleep onset and reduced sleep duration, prompting recommendations to limit screen use before bedtime^2,3^. Nevertheless, a substantial proportion of children sleep in illuminated environments; one study found 39% of kindergarten children slept with room lights on and 50% used a nightlight^4^. These lighting practices, intended for comfort or safe navigation, may suppress nocturnal melatonin and alter circadian timing, negatively impacting sleep^1^. Sleep disturbances affect up to 30% of preschool-aged children^5^ and are associated with long-term effects on health, learning, and development^6^. Thus, evening and nighttime light exposure represents a critical yet often overlooked factor in pediatric sleep health.

While light is essential for vision, it also regulates non-visual physiological processes, including circadian rhythms, melatonin secretion, and alertness. Perceived brightness does not always reflect biological impact because non-visual responses are mediated by intrinsically photosensitive retinal ganglion cells (ipRGCs) that contain melanopsin, distinct from the rods and cones used for vision^7,8^. These ipRGCs project to the suprachiasmatic nucleus in the hypothalamus, the central circadian pacemaker, influencing neuroendocrine and behavioral rhythms^1,7–9^.

Because of this physiology, responses to light depend on timing, intensity, spectral composition, and spatial distribution. Light exposure in the early biological night can delay circadian rhythms, acutely suppress the nocturnal hormone melatonin, and disturb sleep. These effects are amplified with higher intensity and greater short-wavelength (blue) content^10–13^. White lights that appear visually similar may also have dramatically different physiological effects, depending on spectral content^14^. As a consequence, the biological potency of light for non-visual responses is quantified via melanopic equivalent daylight illuminance (melanopic EDI), which weights intensity according to the melanopsin absorption curve^15,16^. In adults, half-maximal circadian phase shifting and melatonin suppression occur with melanopic EDI of approximately 25–70 lx, based on a meta-analysis of multiple studies, including the primary study by Zeitzer et al. that was originally reported in photopic terms^9,17^. Consensus guidelines recommend adults maintain melanopic exposure >250 lx during the day, <10 lx in the evening, and <1 lx at night^18^. Although formal guidelines for children are lacking, evidence suggests they are 5 or more times as sensitive as adults, with significant melatonin suppression and phase delays occurring at melanopic EDI as low as 5 lx^19–22^.

Reducing light exposure before bedtime may promote earlier sleep onset and longer sleep duration. Because children have longer overall sleep needs and an earlier circadian phase than adolescents and adults, they are particularly vulnerable to the effects of evening light exposure. This is likely why interventions advancing sleep times are particularly promising for improving overall sleep health in children, but may also be partly due to early school schedules limiting morning sleep extension^23^. However, minimizing evening light exposure in young children remains challenging in practice. Despite the growing evidence linking light exposure to pediatric sleep disruption, this issue remains largely absent from clinical guidelines and consumer safety standards.

Manufacturers rarely report melanopic or even photopic intensities on nightlight packaging, leaving caregivers and clinicians without clear guidance. Considering the widespread use of nightlights, it is surprising that their biological potency has not been systematically assessed. Understanding how the melanopic intensities of nightlights compare to physiologically relevant thresholds is critical for developing evidence-based recommendations that support pediatric sleep and circadian health.

## Results

**Fig. 1:**
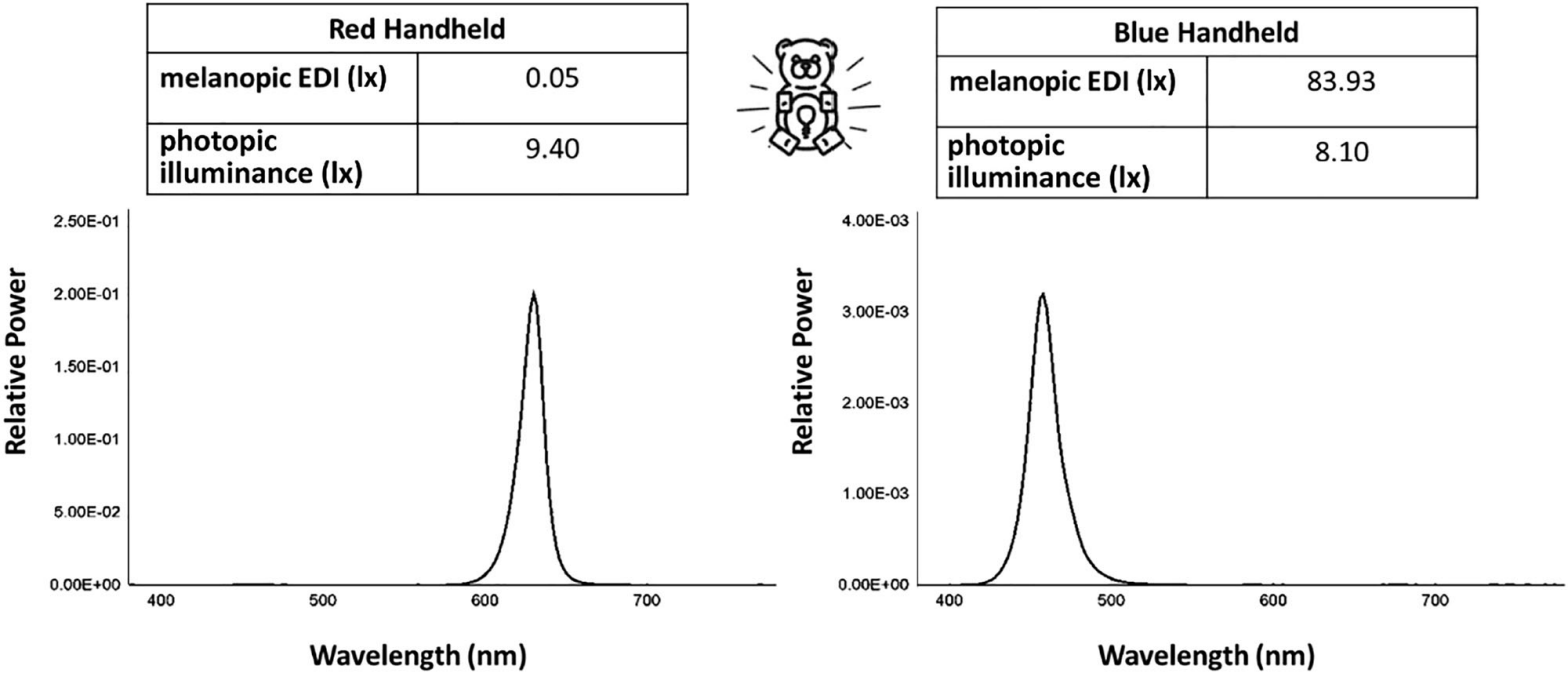
Spectral power distributions (SPDs) for the same nightlight model in two different color options. SPDs for the same model nightlight in red (left) and blue (right) are shown, along with corresponding melanopic and photopic illuminances under typical-use conditions. Though visual brightness (photopic illuminance, lx) is similar, biological potencies (melanopic EDI, lx) are significantly different. The red version of this handheld nightlight is also well below melanopic EDI thresholds, whereas the blue version is not. Further details on these two devices may be found in Tables S1–S3, for nightlights 1 and 2.

**Fig. 2:**
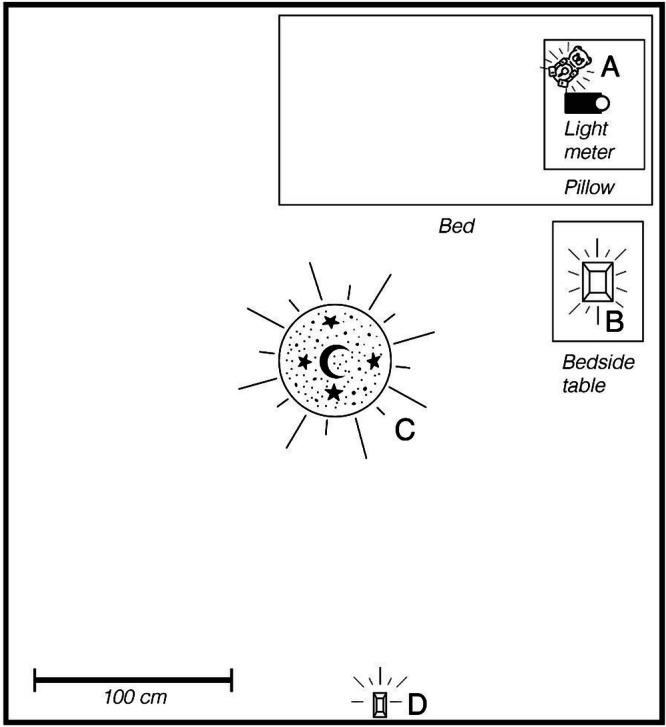
Schematic diagram of nightlight positions under bedroom (typical) use conditions. The relative distance between the light meter (on the pillow) and the nightlights (positioned throughout the room) during measurement is illustrated. Device categories (distances) include: **A** handhelds (15 cm), **B** tabletop lamps (92 cm), **C** projectors (180 cm, measured at ceiling), and **D** wall plug-ins (274 cm).

Based on spectral irradiance measurements (see Fig. 1 for examples), melanopic EDI values were derived and examined across device categories, along with comparisons to known circadian disruption thresholds. Results are presented for both laboratory measurements, representing source-level, maximal ocular exposure (“worst-case”), and for simulated bedroom measurements (Fig. 2), reflecting more typical nighttime use.

### Laboratory (source-level/maximal exposure)

Table S1 provides melanopic EDI values derived from measurements obtained at 2 mm from the light-emitting surface, representing maximal ocular exposure. Across all devices and settings, melanopic EDI values spanned several orders of magnitude, from 0.09 lx to over 100,000 lx, with a median (IQR) of 155.49 (22.22, 516.04) lx. These values reflect source-level measurements, not those at typical viewing distances, but are included to provide full spectral characterization of each nightlight.

A Kruskal–Wallis test confirmed significant differences in melanopic EDI between groups (*χ*²(3) = 22.81, *p* < 0.0001). Post-hoc Dunn’s tests showed handhelds (24.38 (1.27, 140.30) lx) had significantly lower melanopic EDI than projectors (458.31 (264.94, 601.80) lx) (*p* < 0.0001); all other comparisons were not statistically significant (tabletop lamps and wall plug-ins were 116.40 (30.67, 501.63) and 189.65 (6.08, 856.62) lx, respectively) (*p* ≥ 0.16). Fligner–Killeen tests also indicated differences in variance across categories (*χ*²(3) = 16.79, *p* = 0.0008).

In terms of biological relevance, we examined the percent of devices and settings meeting and/or falling below various recommendations (Table 1). Overall, 3/24 devices (13%) met nighttime recommendations (<1 lx melanopic EDI), and 63/79 total settings (80%) failed to meet even the least conservative evening recommendations in adults (<10 lx melanopic EDI) under these maximal, source-level exposures. Only three products remained entirely below 5 lx, the lowest level associated with phase shifting and melatonin suppression in children^21,22^. These were all red-light devices (2 handhelds, 1 wall plug-in) with sufficient visual brightness (13–78 photopic lx) to allow basic nighttime navigation. For the remaining lights with at least one setting falling below the 5 lx melanopic EDI threshold, those lower levels were primarily met with red-appearing light and/or by being on the dimmest available setting (Table S1). While perhaps rare, it is not impossible that children could put their eyes up to these nightlights if placed within reach, making these source-level data relevant for extreme behavior scenarios.

**Table 1:** The proportion of nightlights and settings meeting and failing to meet benchmarks within each device category under both a) laboratory (source-level/maximal exposure) and b) bedroom (typical exposure) conditions

Device type	% devices meeting all recommendations (<1 lx melanopic EDI) *across all settings*	% devices failing to meet adult nighttime recommendations (<1 lx melanopic EDI) *for at least one setting*	% settings <5 lx melanopic EDI (known pediatric thresholds)	% settings with <5 lx melanopic EDI (pediatric threshold) AND >5 photopic lx (for safe navigation)
a) Laboratory (source-level/maximal)				
Handhelds (*n* = 7 devices/24 settings)	29%	71%	33%	25%
Tabletops (*n* = 4 devices/14 settings)	0%	100%	21%	7%
Projectors (*n* = 8 devices/31 settings)	0%	100%	3%	0%
Wall Plug-ins (*n* = 6 devices/10 settings)	17%	83%	20%	20%
b) Simulated Bedroom (typical use)				
Handhelds (*n* = 7 devices/24 settings)	29%	71%	46%	17%
Tabletops (*n* = 4 devices/14 settings)	25%	75%	71%	0%
Projectors (*n* = 8 devices/31 settings)	63%	38%	77%	6%
Wall Plug-ins (*n* = 6 devices/10 settings)	50%	50%	90%	0%

### Bedroom (typical use)

Table S2 presents melanopic EDI values measured in a simulated bedroom environment, reflecting ecologically valid nighttime exposure. In contrast to laboratory results, bedroom measurements showed a much narrower range, from 0 to 907.76 lx, with a median (IQR) of 0.72 (0.07, 6.55) lx. Median (IQR) for each category included: handhelds: 4.33 (0.09, 21.48) lx; tabletop lamps: 1.70 (0.54, 4.31) lx; wall plug-ins: 0.27 (0.03, 0.98) lx; and projectors: 0.22 (0.08, 2.33) lx. Four nightlight settings (from three products) produced 0 lx melanopic EDI on at least one setting, despite being visibly illuminated; however, only two of these products met nighttime melanopic EDI recommendations across all available settings.

Though numerical differences appeared across categories, a Kruskal–Wallis test found no significant differences in the distribution of medians (*χ*²(3) = 6.12, *p* = 0.106); however, a Fligner–Killeen test revealed significant differences in variability (*χ*²(3) = 16.79, *p* < 0.001). Projectors in particular showed some high outliers, likely explained by setup differences. Plug-in projectors, plugged in across the room and reflected off the ceiling (labeled “P” in Table S2), had lower melanopic EDI (0.08 (0.07, 0.30) lx), whereas tabletop projectors, placed closer to the bed and reflected off the ceiling (labeled “B” in Table S2), showed higher melanopic EDI values (1.78 (0.26, 46.86) lx), likely due to proximity to the eyes and stray light from the source.

The percent of devices and settings meeting and/or falling below various recommendations for each nightlight category in the simulated bedroom environment is reported in Table 1. In total, less than half of devices (10/24, 42%) met all nighttime recommendations (<1 lx melanopic EDI) across settings, and 15/79 total settings (19%) failed to meet even the least conservative evening recommendations in adults (<10 lx melanopic EDI). Only 6/79 settings (8%) support both circadian health and visual stimulation (melanopic EDI < 5 lx and photopic >5 lx), including four settings from three devices in the handheld category and the remaining two coming from the same tabletop projector.

## Discussion

This study is the first to systematically quantify the biological potency of commercially available nightlights using melanopic equivalent daylight illuminance (EDI) metrics. Our findings reveal that a substantial proportion of nightlights exceed thresholds known to impact circadian physiology, even under typical-use conditions.

While the maximal exposures recorded in laboratory conditions may represent source-level, worst-case scenarios (e.g., holding a bright nightlight immediately up to the eye), the simulated bedroom measurements suggest that more typical usage still often results in melanopic EDI levels that exceed nighttime recommendations and thresholds^18,21,22^. At the lowest exposure levels, measurements approach the practical noise floor of the spectroradiometer, and values reported as zero reflect signals below the detection threshold under the measurement conditions rather than a confirmed absence of light emission^24^. Yet, such low levels represent the minority of nightlight settings, which is particularly concerning given accumulating evidence that children’s circadian systems are more sensitive to light than adults (and even adults may be more sensitive than presumed^24^); thus, the actual physiological effects of nightlights may be even greater than these data suggest. Importantly, although we did not explicitly evaluate compliance with ICNIRP or toy optical radiation standards, all measured nightlight intensities were far below levels associated with photochemical retinal hazard, indicating minimal risk from blue-light exposure under typical usage conditions.

The variation observed across nightlight types highlights two important considerations for pediatric sleep health. First, spectral composition plays a crucial role: red-hued nightlights consistently showed the lowest theoretical biological potency, which is largely due to reduced ipRGC activation at longer wavelengths^1^. Second, placement and distance from the child’s eye are critical determinants of actual exposure, due to the inverse square law of light propagation and the effects of room reflectance^25^. Our bedroom simulations also underscore that wall plug-in nightlights and projectors (when positioned farther from the bed) generally produce safer light levels, although exceptions exist. In contrast, handheld nightlights, despite often having lower maximum intensity, pose a greater risk of sleep and circadian disruption because they are typically used close to the eyes.

Balancing visual needs and circadian health remains a challenge. Children with a fear of the dark are also at risk for poor sleep quality^26^. Thus, eliminating nightlights from the bedroom altogether may be counterproductive. Instead, the goal should be to provide nightlights that allow children to see but without eliciting non-visual physiological responses to light. Nightlights that provide sufficient photopic illumination for safe navigation typically exceed melanopic thresholds for circadian disruption^18^. Conversely, those that maintain low melanopic exposure often fail to provide adequate light for practical use. However, our data show that some products can strike this balance, suggesting feasible design improvements.

From a clinical perspective, pediatricians should counsel families on the importance of selecting nightlights with lower melanopic content, particularly red or amber lights at the dimmest settings. Placement recommendations include positioning lights away from direct line-of-sight and increasing the distance from the child’s bed, out of reach, when possible. Additional strategies to reduce reliance on nightlights, such as comfort objects or consistent bedtime routines, may also be beneficial.

The absence of standardized labeling or regulatory guidance on nightlight spectral and intensity characteristics is a notable gap. Manufacturers rarely report melanopic or even photopic illuminance, leaving caregivers without critical information to make informed decisions. Given the widespread use of nightlights^4^ and the known heightened sensitivity of children’s circadian systems^19–22^, establishing clinical guidelines, regulatory standards, and clear product labeling is essential to supporting healthy pediatric sleep and circadian health.

Our light measurement data raise practical concerns about the health and safety impacts of nightlights. Future research should empirically test the impact of nightlights with varying melanopic EDI levels on melatonin secretion, sleep timing, and behavioral outcomes in children, including real-world use with handheld lights. Long-term studies are also needed to evaluate the chronic effects of low-level nocturnal light exposure in pediatric populations.

## Methods

This study involved measuring the spectral irradiance of a range of commercially available children’s nightlights under both laboratory and ecologically valid conditions. Our goal was to calculate melanopic equivalent daylight illuminance (melanopic EDI) and evaluate each nightlight’s theoretical biological potency relative to established circadian thresholds.

### Nightlight selection

We selected 25 nightlights marketed for use in children, representing different form factors and spectral profiles to ensure broad generalizability. Specific products were chosen based on popularity and consumer ratings from major online retailers between 2022 and 2024, reflecting typical consumer purchasing behavior rather than a statistically representative sampling of all available products. Nightlights were grouped into four categories that varied according to typical bedroom placement and use: (1) battery-operated or rechargeable handhelds (*n* = 7); (2) tabletop lamps, light boxes, or bare bulbs (*n* = 4); (3) Plug-in or tabletop projectors reflecting light off ceilings or walls (*n* = 8); and (4) flush-mount wall plug-ins (*n* = 6). Some devices (*n* = 4) could fit into multiple categories; in such cases, we used the configuration closest to the bed to capture the highest potential ocular exposure.

### Light measurements

Each nightlight was measured in both the laboratory and a simulated bedroom environment. Laboratory conditions included source-level measurements that characterized maximal emission (2 mm from the emitting surface). These measurements provide standardized, reproducible data across nightlights, though they do not reflect typical viewing behavior. Simulated bedroom measurements were taken to approximate typical use under more ecologically valid conditions, with devices positioned according to common practices and the meter at the level of the eye in bed, capturing more realistic exposure scenarios.

Spectral power distributions (power at each wavelength in the visible spectrum; examples in Fig. 1) and irradiance (total power per unit area) measurements were obtained using a PR-670 spectroradiometer (Photo Research, North Syracuse, NY; Model 2900-0087-40, Serial No. 67200301). The instrument was factory calibrated by the manufacturer (Novanta Corporation, JADAK) on December 21, 2023, with calibration traceable to National Institute of Standards and Technology (NIST) standards. Calibration included wavelength accuracy and absolute spectral sensitivity, with a stated color accuracy of CIE 1931 x,y ± 0.0015, and the device was within its certified calibration period at the time of all measurements. Three measurements were averaged for each nightlight setting in both laboratory and bedroom environments, all performed in complete darkness (confirmed via undetectable ambient light values). A Konica Minolta T-10A illuminance meter (Konica Minolta Inc., Tokyo, Japan) was used as a secondary device to cross-check relative photopic illuminance values derived from spectroradiometric data, particularly at lower light levels; because illuminance meters may exhibit spectral mismatch for narrowband or colored sources, these values were interpreted qualitatively, while all biologically relevant metrics were derived from calibrated spectroradiometric measurements. At very low irradiance levels approaching the spectroradiometer’s noise floor, measurements were interpreted cautiously; values reported as zero reflect signals below the practical detection threshold under the measurement conditions employed, rather than a confirmed absence of optical output.

For laboratory source-level measurements, the meter was positioned within 2 mm of the light-emitting surface. While this represents a “worst-case” source characterization, it does not reflect typical child behavior. Children, however, may occasionally place a light directly at eye level out of curiosity, and thus, the laboratory measurement remains informative beyond just the advantage of producing standardized, more easily reproducible spectral data across devices.

For the bedroom (335 cm × 366 cm), the sensor approximated eye position in the bed, and nightlights were placed in the bedroom according to typical use (Fig. 2). Handhelds were set up in the bed just below the pillow (15 cm from meter); tabletop lights were positioned on a bedside table (92 cm from meter); and plug-in projectors and flush-mount devices were plugged into an outlet on the wall opposite the bed (274 cm from meter). The meter sensor was placed on a tripod on the bed at the level of the pillow top and directed toward the nightlight, except for tabletop projectors and plug-in projectors, when the meter sensor was instead directed at the ceiling to capture reflected light (180 cm from meter to ceiling).

Color and intensity settings were recorded for each nightlight, and up to six configurations per product were measured to assess the full range of potential physiological impact. For example, several nightlights were measured at red, blue, and white settings at both minimum and maximum intensities (detailed in Table S1).

### Analyses

Alpha-opic illuminances, including melanopic EDI, were derived using the Commission Internationale de l’Éclairage S 026/E:2018 toolbox^16^. Each nightlight setting (*n* = 79 total) was treated as an independent observation for statistical comparisons. All statistical analyses were performed using R version 4.5.0 (R Core Team, 2025)^27^. Descriptive statistics provided means, standard errors (SE), median, and interquartile range (IQR), and the proportion of nightlights and settings that met or exceeded thresholds for circadian health (<1 and 5 lx melanopic EDI) were calculated. The distribution of melanopic EDI data was assessed using the Shapiro–Wilk test, which indicated significant deviation from normality (*p* < 0.0001). Non-parametric methods were therefore employed to assess group differences at the *p* < .05 level. Specifically, the Kruskal–Wallis test compared median melanopic EDI values across nightlight categories, and Dunn’s test with Bonferroni correction was employed for post-hoc pairwise comparisons. In addition, the Fligner–Killeen test compared variances across nightlight types. We acknowledge that rankings and measurements are context-specific, based on the tested units under defined conditions; product availability, specifications, or light sources may change over time. In addition, statistical analyses were primarily descriptive, aimed at summarizing melanopic EDI distributions and identifying patterns across nightlight types. While formal hypothesis testing was performed to compare medians and variances, we emphasize that the primary purpose of these analyses is not to establish definitive inferential conclusions, but rather to provide a structured and transparent framework for comparing products and protecting against potential bias in reporting.

## Supplementary information

Supplementary Information

## Data Availability

All data generated or analyzed during this study are included in this published article and its supplementary information files.
