# Supplementary material for: Quantifying the biological impacts of nightlights: implications for sleep and circadian health in children
Source: NPJ Biol Timing Sleep. 2026 Apr 2;3:14. doi: 10.1038/s44323-026-00072-6 (PMC13046988; doi:10.1038/s44323-026-00072-6)
Supplement: Supplementary file 1 — Supplementary Information [file 44323_2026_72_MOESM1_ESM.pdf]

Supplementary Materials. Glickman, Biological Impacts of Nightlights.

Table S1. Laboratory Measurements (source level)

Nightlight	Color/Intensity Setting	Melanopic EDI (lx)	Photopic Illuminance (lx)	s-cone	m-cone	l-cone	rho	Irradiance (W/m2)	CCT (K)
**A. Battery-Operated or Rechargeable Portables and Handhelds**									
**1**	blue	538.04	51.94	965.65	149.58	77.61	401.68	1.03	-
**2**	red	0.44	77.99	0.37	21.07	95.58	1.79	0.37	-
**3.1**	red, low	0.09	7.04	0.04	1.91	8.63	0.22	0.03	-
**3.2**	red, high	0.24	42.42	0.32	11	52.18	0.91	0.21	-
**3.3**	blue, low	28.89	3.2	43.22	8.55	4.54	21.62	0.05	-
**3.4**	blue, high	263.04	29.84	390.9	78.82	41.76	197.24	0.46	-
**3.5**	white, low	16.23	23.55	19.48	22.09	22.91	18.04	0.07	5356
**3.6**	white, high	155.01	221.57	183.36	208.08	215.87	171.33	0.67	5653
**4.1**	red, low	1.55	5.61	0.95	2.03	6.87	1.37	0.03	-
**4.2**	red, high	1.79	48.34	1.21	12.91	59.41	2.27	0.25	-
**4.3**	blue, low	13.38	1.86	20.05	4.28	2.45	10.16	0.03	-
**4.4**	blue, high	118.21	14.83	186.13	36.28	20.15	89.2	0.22	-
**4.5**	white, low	19.87	13.06	21.12	13.04	14.09	17.87	0.06	7740

**4.6**	white, high	177.54	122.25	190.31	120.72	130.88	161.48	0.59	7117
**5.1**	multi-1	46.21	47.72	58.75	39.3	52.36	41.52	0.19	2625
**5.2**	multi-2	78.49	32.03	100.2	42.66	33.78	67.46	0.18	-
**5.3**	multi-3	135.4	110.99	166.61	101.02	120.96	119.94	0.46	4386
**6.1**	red, low	0.21	5.73	0.2	1.6	7.01	0.27	0.03	-
**6.2**	red, high	0.37	45.25	0.21	11.72	55.44	1.16	0.26	-
**6.3**	blue, low	40.21	5.76	72.73	12.51	7.66	30.58	0.08	-
**6.4**	blue, high	400.51	53.44	714.68	124.07	71.37	304.59	0.8	-
**6.5**	white, low	19.87	28.92	22.18	26.91	28.23	22.13	0.09	5314
**6.6**	white, high	190.71	256.14	212.51	251.05	257.85	210.32	0.81	5707
**7**	red	0.19	13.05	0.31	3.48	16.02	0.39	0.06	-
**Mean**		**93.60**	**52.61**	**140.48**	**54.36**	**58.48**	**78.90**	**0.29**	**5487.25**
**SE**		**28.39**	**13.44**	**48.94**	**14.20**	**13.49**	**22.30**	**0.06**	**555.16**
**Median**		**24.38**	**30.94**	**32.70**	**21.58**	**37.77**	**21.88**	**0.20**	**5504.50**
**IQR**		**139.03**	**40.77**	**183.25**	**73.98**	**60.21**	**78.90**	**0.40**	**977.50**
**B. Tabletop Light Boxes, Lamps, and Bulbs**									
**1**	orange	2110.6	17962	593.61	10904.99	19158.39	3767.49	61.11	1651
**2**	white	25.5	32.75	29.36	31.29	32.01	27.15	0.1	6073
**3.1**	multi-1	1.57	272.11	0.74	75.24	332.57	6.66	1.3	-

**3.2**	multi-2	0.33	31.48	0.27	8.92	38.45	0.92	0.14	-
**3.3**	multi-3	3463.96	413.78	4688.25	1068.47	569.11	2603.12	5.92	-
**3.4**	multi-4	374.21	44.39	512.61	114.92	61.17	280.98	0.64	
**3.5**	multi-5	5047.14	2451.36	4357.68	3273.85	2422.37	4634.5	10.43	-
**3.6**	multi-6	544.1	265.35	471.44	353.2	262.51	499.93	1.13	-
**4.1**	multi-1	127.58	275.35	89.4	220.31	279.91	148.31	0.84	2882
**4.2**	multi-2	1.71	3.45	1.49	2.75	3.53	1.9	0.01	2864
**4.3**	multi-3	155.49	58.5	160.38	87.7	58.43	136.48	0.29	-
**4.4**	multi-4	70.31	83.15	22.55	80.53	81.78	79.19	0.25	4473
**4.5**	multi-5	105.21	91.76	58.96	98.76	89.48	109.6	0.3	6747
**4.6**	multi-6	46.18	76.03	17.01	57.76	80.49	51.72	0.27	2128
**Mean**		**862.42**	**1575.82**	**785.98**	**1169.91**	**1676.44**	**882.00**	**5.91**	**3831.14**
**SE**		**419.38**	**1271.62**	**427.08**	**783.90**	**1355.04**	**418.98**	**4.32**	**746.90**
**Median**		**116.40**	**87.46**	**74.18**	**93.23**	**85.63**	**123.04**	**0.47**	**2882.00**
**IQR**		**470.96**	**226.62**	**483.92**	**257.85**	**260.29**	**411.90**	**1.00**	**2777 .00**
**C. Plug-In and Table-Top Projectors**									
**1**	multi	423.34	483.26	394.85	450.72	489.98	416.63	1.67	4839
**2.1**	red	2.47	463.17	0.39	122.75	568.67	10.23	2.23	-
**2.2**	blue	2210.46	213.38	3792.89	617.15	318.46	1646.39	4.12	-

**2.3**	white	255.85	731.11	237.94	583.37	727.81	340.28	2.04	3112
**3.1**	red	398.27	1215.57	310.5	900.38	1248.79	502.99	3.61	2426
**3.2**	blue	489.55	777.39	486.3	683.23	777.5	531.66	2.29	4076
**3.3**	green-blue	629.91	1266.09	518.39	1089.78	1249.46	745.1	3.33	3837
**3.4**	green	412.38	901.09	312.45	760.43	892.09	499.32	2.34	3583
**3.5**	pink	494.76	1272.84	491.35	943.09	1322.25	574.46	3.9	2374
**3.6**	yellow	464.28	1796.81	231.06	1344.41	1816.1	665.01	4.63	2577
**4.1**	orange	327.16	1012.82	255.79	741.95	1044.4	412.24	3.08	2351
**4.2**	orange with black	123.68	687.92	48.4	458.84	718.3	191.79	2.05	1959
**4.3**	orange with white	274.02	895.13	199.18	653.74	921.56	353.24	2.7	2346
**4.4**	green	342.42	826.8	259.79	673.33	826.52	420.66	2.22	3201
**4.5**	blue	493.48	1001.01	445.92	836.27	1002.23	571.06	2.77	3449
**4.6**	blue	537.32	1010.2	510.55	860.09	1008.82	610.23	2.81	3686
**5.1**	red, low	24.56	5659.45	14.39	1529.07	6935.03	123.89	27	-
**5.2**	red, high	134.18	27198.23	83.52	7219.68	33366.19	589.97	133.97	-
**5.3**	green, low	94.9	156.7	3.72	172.64	129.35	139.18	0.27	7463
**5.4**	green, high	10848.57	18168.32	258.36	20009.41	14972.02	16021.28	30.45	7451
**5.5**	blue, low	20407.84	1821.09	42084.42	5457.64	2832.2	15251.75	42.2	-

**5.6**	blue, high	108067.12	9783.13	220311.38	29076.96	15132.56	80771.17	223.09	-
**6.1**	red	140.5	590.39	130.1	385.14	622.4	192.33	2.04	1830
**6.2**	blue	458.31	456.99	670.38	427.59	469.63	424.63	1.74	7373
**6.3**	white	146.91	455.31	137.8	360.47	452.65	198.01	1.23	3086
**7.1**	green	26007.42	10818.56	2957.18	17500.7	10787.63	25921.62	38.02	12237
**7.2**	blue, low	29094.65	1881.39	92991.21	6793.82	3674.72	21727.73	86.56	-
**7.3**	blue, high	118132.59	7652.13	375940.65	27595.49	14922.92	88214.31	350.37	-
**8.1**	multi-1	287.98	204.13	324.42	192.88	222.67	260.88	1	4886
**8.2**	multi-2	565.64	220.6	816.77	273.6	246.66	472.31	1.45	-
**8.3**	multi-3	573.68	218.16	836.1	273.02	244.9	476.97	1.46	-
**Mean**		**10414.97**	**3220.62**	**24066.33**	**4160.89**	**3869.18**	**8363.78**	**31.83**	**4197.24**
**SE**		**5115.51**	**1075.03**	**14003.04**	**1436.01**	**1281.79**	**3849.03**	**13.58**	**552.37**
**Median**		**458.31**	**901.09**	**324.42**	**741.95**	**921.56**	**499.32**	**2.77**	**3449.00**
**IQR**		**336.86**	**1335.74**	**528.46**	**997.59**	**1794.83**	**358.30**	**13.78**	**2413.00**
**D. Wall Plug-Ins**									
**1**	blue	1247.02	124.61	2142.59	351.4	183.17	930.43	2.33	-
**2.1**	red	7.71	918.16	3.79	245.99	1125.38	23.18	4.54	-
**2.2**	orange	31.03	1237.91	1.82	750.36	1323.47	125.36	2.48	1730
**3**	blue	908.12	90.71	1600.18	256.14	133.54	679.29	1.72	-

**4**	red	0.3	55.13	0.23	15.3	67.42	1.32	0.25	-
**5.1**	red	0.46	72.71	0.31	19.85	89.13	1.71	0.34	-
**5.2**	blue	348.27	43.76	445.21	110.47	59.14	263.17	0.59	-
**5.3**	white	702.13	1513.88	565.66	1223.67	1532.15	827.83	4.55	3012
**6.1**	white, low	5.53	11.89	4.02	9.66	12.01	6.56	0.04	3038
**6.2**	white, high	4688.1	9434.23	3574.71	7739.35	9544.35	5401.91	28.78	3109
**Mean**		**793.87**	**1350.30**	**833.85**	**1072.22**	**1406.98**	**826.08**	**4.56**	**2722.25**
**SE**		**455.47**	**915.75**	**387.23**	**750.73**	**923.35**	**521.27**	**2.74**	**331.38**
**Median**		**189.65**	**107.66**	**224.62**	**251.07**	**158.36**	**194.27**	**2.03**	**3025**
**IQR**		**850.55**	**1098.45**	**1339.24**	**608.12**	**1201.10**	**779.98**	**3.62**	**364.25**

For each category of nightlight (A-D), the first column includes whole numbers to identify the nightlight and decimal places to represent separate settings for that light. Each cell contains an average of three values derived for each device setting based on spectral irradiance measures using the PR-670 spectroradiometer (with color appearance and intensity setting noted), including alpha-opic illuminances; photopic lux and irradiance (W/m^2); and CCT (in degrees Kelvin), where applicable. Means, standard error (SE), median and interquartile range (IQR) are provided for each nightlight category. Shaded rows highlight settings for which consensus-based nighttime recommendations (melanopic EDI <1 lux) have been met.

Table S2. Simulated Bedroom Measurements (typical use exposure)

A. Battery-Operated or Rechargeable Portables and Handhelds			B. Tabletop Light Boxes, Lamps, and Bulbs			C. Plug-In and Table-Top Projectors			D. Wall Plug-Ins		
**Night-light**	**Melanopic EDI (lx)**	**Photopic Illuminance (lx)**	**Night-light**	**Melanopic EDI (lx)**	**Photopic illuminance (lx)**	**Night-light**	**Melanopic EDI (lx)**	**Photopic Illuminance (lx)**	**Night-light**	**Melanopic EDI (lx)**	**Photopic illuminance (lx)**
**1**	83.93	8.10	**1**	6.12	51.50	**1 (P)**	0.22	0.25	**1**	6.98	0.70
**2**	0.05	9.40	**2**	0.41	0.51	**2.1 (T)**	0	0.95	**2.1**	0.04	0.42
**3.1**	0.09	5.27	**3.1**	0	0.68	**2.2 (T)**	15.47	1.45	**2.2**	0.25	0.58
**3.2**	0.23	51.60	**3.2**	0	0.08	**2.3 (T)**	2.84	8.12	**3**	0.80	0.08
**3.3**	28.32	7.74	**3.3**	8.66	1.03	**3.1 (P)**	0.05	0.16	**4**	0	0.11
**3.4**	257.78	75.90	**3.4**	0.94	0.11	**3.2 (P)**	0.10	0.15	**5.1**	0	.08
**3.5**	8.12	10.29	**3.5**	12.62	6.13	**3.3 (P)**	0.08	0.15	**5.2**	1.04	0.12
**3.6**	77.51	101.40	**3.6**	1.36	0.66	**3.4 (P)**	0.08	0.14	**5.3**	0.29	0.63
**4.1**	0.07	0.26	**4.1**	3.70	7.99	**3.5 (P)**	0.08	0.16	**6.1**	0.02	0.04
**4.2**	0.07	1.85	**4.2**	0.05	0.10	**3.6 (P)**	0.09	0.33	**6.2**	1.83	3.67
**4.3**	.79	0.11	**4.3**	4.51	1.70	**4.1 (P)**	0.07	0.20			
**4.4**	5.08	0.63	**4.4**	2.04	2.41	**4.2 (P)**	0.04	0.12			
**4.5**	0.85	0.56	**4.5**	3.05	2.66	**4.3 (P)**	0.08	0.14			
**4.6**	7.10	4.89	**4.6**	1.34	2.20	**4.4 (P)**	0.02	0.12			
**5.1**	5.18	5.32				**4.5 (P)**	0.05	0.12			
**5.2**	12.19	9.47				**4.6 (P)**	0.06	0.13			
**5.3**	23.55	2.82				**5.1 (T)**	141.03	233.00			

**6.1**	0.03	0.74				**5.2 (T)**	13.57	23.80			
**6.2**	0.05	6.10				**5.3 (T)**	0.72	145.60			
**6.3**	3.58	0.51				**5.4 (T)**	0.29	70.10			
**6.4**	30.44	4.06				**5.5 (T)**	907.76	82.40			
**6.6**	2.13	3.10				**5.6 (T)**	244.89	21.60			
**6.5**	20.79	28.90				**6.1 (T)**	0.18	0.54			
**7**	0.01	0.14				**6.2 (T)**	0.06	0.23			
						**6.3 (T)**	0.37	0.36			
						**7.1 (P)**	1.82	0.72			
						**7.2 (P)**	23.63	1.18			
						**7.3 (P)**	6.98	0.45			
						**8.1 (P)**	0.26	0.18			
						**8.2 (P)**	0.40	0.16			
						**8.3 (P)**	0.34	0.14			
**Mean**	**23.66**	**14.13**	**Mean**	**3.20**	**5.55**	**Mean**	**43.92**	**19.13**	**Mean**	**1.13**	**0.643**
**SE**	**11.19**	**5.27**	**SE**	**1.00**	**3.59**	**SE**	**30.15**	**9.10**	**SE**	**0.68**	**0.35**
**Median**	**4.33**	**5.08**	**Median**	**1.70**	**1.37**	**Median**	**0.22**	**0.25**	**Median**	**0.27**	**0.27**
**IQR**	**21.40**	**8.71**	**IQR**	**3.77**	**2.05**	**IQR**	**2.26**	**1.17**	**IQR**	**0.96**	**0.53**

Melanopic EDI (lx) and photopic illuminance (lx) for each nightlight setting derived from spectral irradiance measures using a PR-670 spectroradiometer in a simulated bedroom environment, by category, including: A) battery-operated or rechargeable portables and handhelds; B) tabletop light boxes, lamps, and bulbs; C) projectors (T=tabletop and P=plug-in), and D) wall plug-ins. Mean, standard error (SE), median and interquartile range (IQR) are also provided for each category. Shaded cells highlight settings for which consensus-based nighttime recommendations (melanopic EDI <1 lx) have been met.

Table S3. Product Details for Nightlight Data

Nightlight	Device Name	Model number	Manufacturer
**A. Battery-Operated or Rechargeable Portables and Handhelds**			
1	GummyGoods Squeezable Night Light (blue)	LPGUM06-V2/LPGUM08	FCTRY, Brooklyn, NY, USA
2	GummyGoods Squeezable Night Light (red)	LPGUM06-V2/LPGUM04	FCTRY, Brooklyn, NY, USA
3	Lumipet Baxter the Bunny	Item 0167	LumieWorld, Zumbrota, MN, USA
4	BrightWorld	8541866736	Shenzhen Little Dragonfly Technology Co., Ltd., Shenzhen, Guangdong, China
5	Toothless Sleeptime Lite Plush Toy	025033417L	Pillow Pets, CJ Products Inc., San Marcos, CA, USA
6	Eternal Moon Nightlight	YQD12	Eternal Home Collection, Sharjah, UAE
7	Minecraft Redstone Block Nightlight	MPN: TX130702-4023	ThinkGeek, Fairfax, VA, USA
**B. Tabletop Light Boxes, Lamps, and Bulbs**			
1	Harth Nite-Nite Light bulb (no lamp shade)	LB-NS-7W	Harth, San Antonio, TX, USA
2	Cloud Island LED Light Box Mountains	DPCI 030-02-9113	Target Corporation, Minneapolis, MN, USA
3	3D Lamp Illusion- Dinosaur	B0CJF3WSD1	Aolalow, Torrance, California, USA
4	Hatch Rest (2nd generation)	HBR4400	Palo Alto, CA, USA
**C. Plug-In and Table-Top Projectors**			

1	Disney 2 Frozen Projectable	45027	Jasco Products Company, LLC, Oklahoma City, OK, USA
2	Moredig Rotating Projector	RCPL-001	Moredig, China
3	Paw Patrol Projectable	30604	Jasco Products Company, LLC, Oklahoma City, OK, USA
4	Jurassic World Projectable	42034	Jasco Products Company, LLC, Oklahoma City, OK, USA
5	Astronaut Galaxy Projector, Star Projector, Nebula Lamp	FlyEagle BL-HJ07	FlyEagle, China
6	Star Master Dream Rotating Projector Lamp	#6917708	China
7	Bliss Lighter Sky Lite Galaxy Projector	900-00047	BlissLights LLC, San Marcos, CA, USA
8	Motion Projectables Northern Lights Night Light	30404	Jasco Products Company, LLC, Oklahoma City, OK, USA
**D. Wall Plug-Ins**			
1	Amerelle Forever Glo Nitelite	71282	Amerelle, Milwaukee, Wisconsin, USA
2	Emagine AutoSensor Amber	CO11B	Emagine A, China
3	Elsent LED Blue	3M28VC9	Elsent, Amazon.com, Seattle, WA, USA
4	SomiLight Red Nightlight	43224-22567	SomniLight, Shawnee, Kansas, USA
5	"Briignite Night Lights Plug Into Wall, Color Changing Night Light for Kids, RGB Night Lights	ABRGY01909US-2	Briignite, China
6	Jasco Dimmable Nightlight	45125	Jasco Products Company, LLC, Oklahoma City, OK, USA

Nightlight numbers correspond to those for which data is reported within each category of Tables S1 and S2. All information is current as of the writing of this manuscript; however, product availability, specifications, and internal light sources are subject to change over time.
